# Effectiveness of a Novel Liposomal Methylglyoxal–Tobramycin Formulation in Reducing Biofilm Formation and Bacterial Adhesion

**DOI:** 10.3390/antibiotics14010003

**Published:** 2024-12-24

**Authors:** Wed Alluhaim, Manal M. Alkhulaifi, Raghad R. Alzahrani, Bahauddeen M. Alrfaei, Alaa Eldeen B. Yassin, Majed F. Alghoribi, Ahlam M. Alsaadi, Ahmed I. Al-Asmari, Ahmed J. Al-Fahad, Rizwan Ali, Naif M. Alhawiti, Majed A. Halwani

**Affiliations:** 1 Department of Botany and Microbiology, College of Science, King Saud University, Riyadh 11451, Saudi Arabia; wedd.luhaim@gmail.com (W.A.); raghad.r.alzahrani@gmail.com (R.R.A.); 2 Nanomedicine Department, King Abdullah International Medical Research Center, King Saud Bin Abdulaziz University for Health Sciences, Riyadh 11481, Saudi Arabia; 3 Stem Cells and Regenerative Medicine, King Abdullah International Medical Research Center, King Saud Bin Abdulaziz University for Health Sciences, Riyadh 11481, Saudi Arabia; alrfaeiba@ngha.med.sa; 4 College of Pharmacy, King Abdullah International Medical Research Center, King Saud Bin Abdulaziz University for Health Sciences, Riyadh 11481, Saudi Arabia; yassina@ksau-hs.edu.sa; 5 Infectious Diseases Research Department, King Abdullah International Medical Research Center, King Saud Bin Abdulaziz University for Health Sciences, Riyadh 11481, Saudi Arabia; alghoribima@ngha.med.sa (M.F.A.); alsaadiah@kaimrc.edu.sa (A.M.A.); 6 Special Toxicological Analysis Section, Pathology and Laboratory Department, King Faisal Specialist Hospital and Research Center, Riyadh 11211, Saudi Arabia; aalasmari1@kfshrc.edu.sa; 7 Faculty of Medicine, Alfaisal University, Riyadh 11533, Saudi Arabia; 8 National Center for Biotechnology, Life Science and Environment Research Institute, King Abdulaziz City for Science and Technology (KACST), Riyadh 12354, Saudi Arabia; ajlfahad@kacst.edu.sa; 9 Medical Research Core Facility and Platforms, King Abdullah International Medical Research Center, King Saud Bin Abdulaziz University for Health Sciences, Riyadh 11481, Saudi Arabia; aliri@kaimrc.edu.sa; 10 Department of Clinical Laboratory Sciences, King Abdullah International Medical Research Center, College of Applied Medical Sciences, King Saud Bin Abdulaziz University for Health Sciences, Riyadh 11481, Saudi Arabia; hawitin@ksau-hs.edu.sa

**Keywords:** liposomes, manuka honey, methylglyoxal, tobramycin, biofilm

## Abstract

**Background:** The emergence of multidrug-resistant bacteria presents a significant global health threat. Liposomal antibiotics have shown a potential to improve antibiotic delivery and efficacy. This study aimed to develop liposomes encapsulating tobramycin (TOB) and methylglyoxal (MGO) to enhance TOB activity while reducing bacterial adhesion and biofilm formation. **Methods:** Clinical isolates of *Pseudomonas aeruginosa* and *Klebsiella pneumoniae* were characterized using whole-genome sequencing. Liposomes (Lip-MGO-TOB) were formulated using Manuka honey as a surfactant and loaded with MGO and TOB. Antibacterial activity, biofilm formation, and bacterial cell adhesion assays were performed to compare the efficacy of Lip-MGO-TOB against free TOB. Liposome characterization included analyses of morphology, zeta potential, TOB encapsulation efficiency, and stability under various biological conditions. **Results:** The Lip-MGO-TOB formulation, at a minimum inhibitory concentration (MIC) of 32 µg/mL, reduced the biofilm formation of the *P. aeruginosa* isolate (PA85) by 68%. Conversely, free TOB, at a MIC of 64 µg/mL, achieved only a 21% reduction. For the *K. pneumoniae* isolate (KP57), Lip-MGO-TOB inhibited bacterial adhesion to A549 cells at a lower concentration (256 µg/mL) compared to free TOB (512 µg/mL). Lip-MGO-TOB demonstrated sustained drug release over 24 h under tested conditions and retained over 99% of TOB. **Conclusions:** The Lip-MGO-TOB formulation significantly enhanced TOB activity against resistant bacteria compared to free TOB. Additionally, it provided a stable drug delivery system with controlled drug release. Liposomal TOB represents a promising advancement in combating antibiotic resistance by improving the efficacy and delivery of conventional antibiotics.

## 1. Introduction

Antibiotic discovery has been a groundbreaking milestone in the history of medicine [1]. Since their discovery, antibiotics have revolutionized healthcare by enabling critical medical procedures such as cancer treatment, organ transplantation, open-heart surgery, and infectious disease treatment [1]. Tobramycin (TOB), an aminoglycoside, is widely used to treat infections caused by Gram-negative pathogens, including *Pseudomonas aeruginosa* and *Klebsiella pneumoniae*. However, its systemic application is limited by bacterial resistance and associated toxicity [2]. Antibiotic resistance is ranked as one of the leading public health threats of the 21st century, second only to cardiovascular disease [3]. According to the World Health Organization, it remains a severe public health problem and a priority for research and development [4]. Addressing this urgent issue requires extensive research into new antibiotic delivery strategies to enhance efficacy and overcome resistance [5]. Among these strategies, nanoscaled drug delivery systems, such as liposomal systems, show significant promise in combating multidrug-resistant (MDR) bacteria [6].

Liposomes are versatile nanocarriers with significant potential for treating MDR, biofilm-forming bacteria. Their unique structure and lipid composition enable the development of various liposomal formulations with enhanced pharmacokinetic and pharmacodynamic properties [7]. These spherical vesicles are composed of cholesterol and natural, nontoxic, inert phospholipids, which closely resemble cellular membranes. This similarity facilitates the targeted delivery of encapsulated antibiotics to infected sites, tissues, or pathogens in a controlled and efficient manner [7,8,9]. Recent studies suggest that incorporating honey into nanoparticles can enhance biological interactions, thereby improving therapeutic outcomes [10].

Manuka honey (MH) has been used as a natural wound-healing agent for over 2000 years, and several clinical trials have explored its therapeutic benefits [11]. MH is a monofloral honey derived from the nectar of Manuka tree flowers [12] and is renowned for its unique antibacterial activity [11]. This activity is attributed to antimicrobial compounds, including surfactants and peptides [13]. A primary factor driving MH’s antibacterial activity is its high concentration of methylglyoxal (MGO) [14]. MGO, an electrophile and natural metabolite associated with glycolytic intermediates such as dihydroxyacetone phosphate, induces cell death at elevated concentrations [15,16]. The MGO content in MH is measured using the Unique Manuka Factor (UMF) classification system, wherein higher UMF values correspond to higher MGO concentrations [17,18]. This study aimed to develop and characterize a novel liposomal formulation (Lip-MGO-TOB) combining TOB and MGO to enhance the efficacy of TOB against clinically resistant bacterial isolates. Additionally, we assessed the formulation’s antibacterial, antibiofilm, and bacterial adhesion inhibition properties. Whole genome sequencing (WGS) was performed to comprehensively analyze the clinical isolates, providing insights into their resistance mechanisms and the potential impact of the developed liposomes.

## 2. Results

### 2.1. Characterization of the Lip-MGO-TOB Formulation

#### 2.1.1. Size, Polydispersity Index (PDI), Encapsulation Efficacy (EE), and Drug Loading Capacity (DLC) of Lip-MGO-TOB

The properties of the Lip-MGO-TOB formulation are summarized in Table 1. The PDI indicated a slightly heterogeneous particle size distribution within the liposomal population. The formulation achieved a high TOB EE, which can be attributed to its particle size (582 nm), remaining within the acceptable nanoscale range. The zeta potential of the loaded liposomes was +26 mV, indicating good stability.

#### 2.1.2. Morphology of the Lip-MGO-TOB Formula

**Figure 1:**
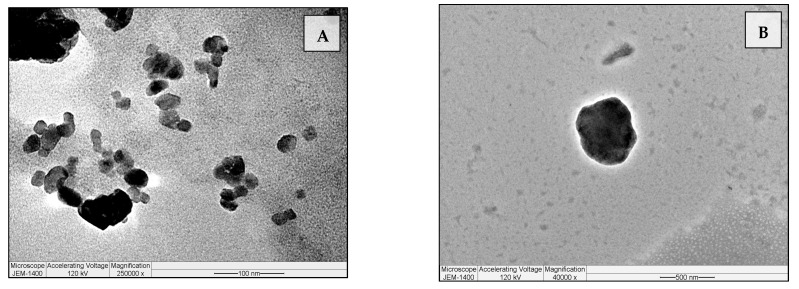
TEM images of the Lip-MGO-TOB formulation. (**A**) The Lip-MGO-TOB formulation showing both single and aggregated particles at 25,000× magnification. (**B**) A zoomed-in view of a spherical liposome at 40,000× magnification.

Figure 1 illustrates the morphology of the Lip-MGO-TOB formulation. The samples displayed a spherical shape with sizes ranging from approximately 20 to 500 nm, consistent with the PDI results. The dark regions observed within the liposomes likely represent the encapsulated antibiotics. The aggregation of liposomes (Figure 1A) may be attributed to the lyophilization process and the hydrophobic nature of the lipids used in the formulation.

#### 2.1.3. Stability of the Lip-MGO-TOB Formulation Under Different Biological Conditions

**Figure 2:**
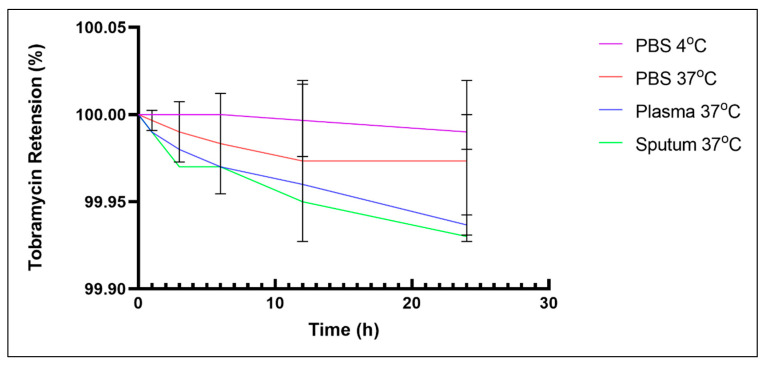
Stability of Lip-MGO-TOB in plasma, sputum, and PBS.

The stability of the Lip-MGO-TOB formulation was evaluated by measuring the percentage of TOB retention after exposure to various biological conditions over 24 h (Figure 2). The formulation demonstrated high stability as a drug delivery system, retaining all encapsulated TOB in phosphate-buffered saline (PBS) at 4 °C for 24 h. The retention rate remained at 100% during the first hour and gradually declined over the 3–24 h period. Overall, the Lip-MGO-TOB formulation exhibited consistent drug release across all tested conditions during the 24-h evaluation.

### 2.2. Biological Assays

#### 2.2.1. Bacterial Clinical Isolates and Susceptibility Tests

Based on the antimicrobial profile (Table 2), *K. pneumoniae* was identified as pandrug-resistant (PDR). PDR isolates exhibited high resistance to all tested antibiotics. However, KP45 showed intermediate resistance to gentamicin. Additionally, KP57 had the highest resistance score (resistance score = 32/32) among all the tested antibiotics. *K. pneumoniae* clinical isolates were resistant to all examined antibiotics, including those belonging to the third and fourth generations of cephalosporins, beta-lactamase inhibitors, carbapenems, aminoglycosides, and fluoroquinolone classes. Colistin was the only effective antibiotic against these resistant isolates.

#### 2.2.2. Biological Activity of TOB and the Lip-MGO-TOB Formulation

##### Checkerboard Assay for Free TOB and MGO

A checkerboard assay was performed to evaluate the combined effects of free TOB and MGO. The synergistic interaction between TOB and MGO was confirmed by a fractional inhibitory concentration index (ΣFICI) value of 0.2578 against the tested *P. aeruginosa* isolate. The MIC of TOB alone was 256 µg/mL, which decreased significantly to 2 µg/mL when combined with MGO. Similarly, the MIC of MGO was reduced from 39 to 9.76 µg/mL when combined with TOB.

##### MIC, MBC, and Antibiofilm Activity

The MIC and MBC results demonstrated that the Lip-MGO-TOB formulation enhanced TOB’s antibacterial activity. Free TOB showed twofold higher MICs (1024–1064 µg/mL) and MBCs (2048–2128 µg/mL) compared to the Lip-MGO-TOB formulation, which exhibited MICs of 512–532 µg/mL and MBCs of 1024–1064 µg/mL (Table 3).

**Figure 3:**
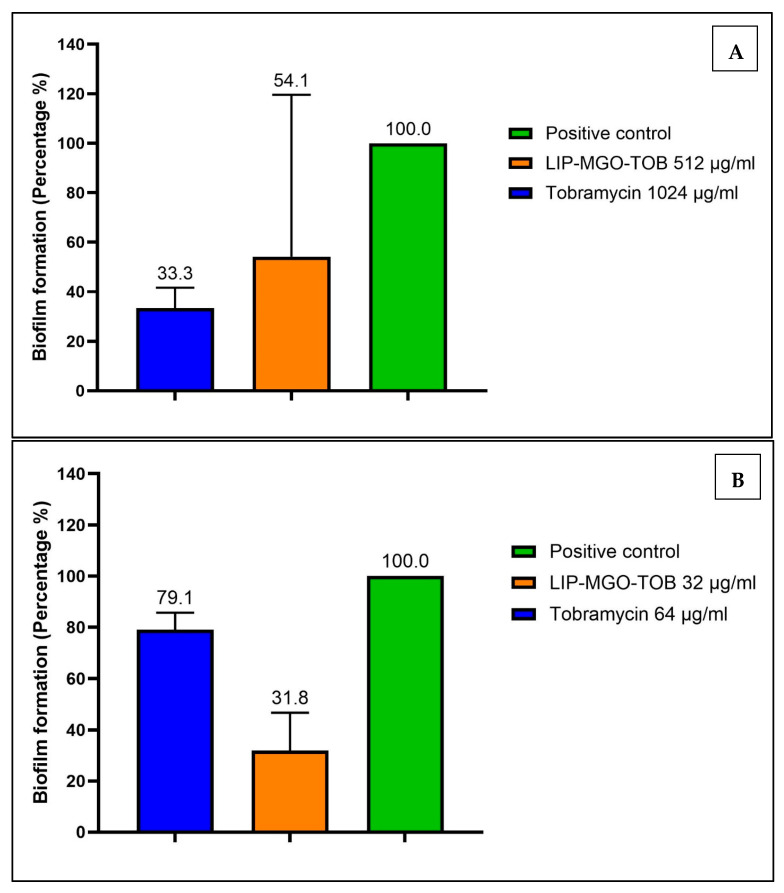
Percentage of biofilm reduction achieved by Lip-MGO-TOB (orange bar) and tobramycin (blue bar) against (**A**) *K. pneumoniae* (KP57) and (**B**) *P. aeruginosa* (PA85). The green bar represents the untreated biofilm (positive control).

The Lip-MGO-TOB formulation was more effective than free TOB in inhibiting the biofilm formation of *K. pneumoniae* (KP57) and *P. aeruginosa* (PA85) at twofold lower concentrations. The MIC of Lip-MGO-TOB inhibited the biofilm of *K. pneumoniae* (KP57) by 45% (Figure 3A). In comparison, free TOB inhibited 66% of KP57’s biofilm, although this was significantly less effective than the untreated control (*p* = 0.036). For *P. aeruginosa* (PA85), the MIC of Lip-MGO-TOB significantly inhibited biofilm formation by 68% (*p* = 0.0001), compared to only 21% inhibition achieved by free TOB at a twofold higher concentration (Figure 3B).

#### 2.2.3. Bacterial Cell Adhesion Inhibition by the Lip-MGO-TOB Formula

**Figure 4:**
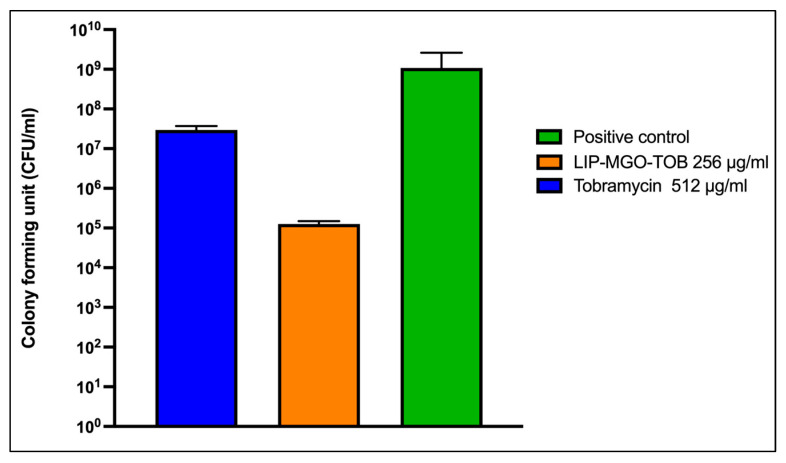
Activity of sub-MIC concentrations of free tobramycin and the Lip-MGO-TOB formulation in preventing *K. pneumoniae* (KP57) adhesion to A549 cells after 3 h of incubation. The green bar represents untreated A549 cells (positive control).

The effect of sub-MIC concentrations of free TOB (512 µg/mL) and the Lip-MGO-TOB formulation (256 µg/mL) on the adhesion of *K. pneumoniae* (KP57) to A549 cells was evaluated (Figure 4). The free TOB (sub-MIC) reduced bacterial adhesion by twofold compared to the positive control. Conversely, the Lip-MGO-TOB formulation achieved a fourfold reduction in bacterial adhesion compared to the positive control despite using a twofold lower TOB concentration.

### 2.3. WGS and Bioinformatics Analysis

#### Multilocus Sequence Typing (MLST)

MLST analysis identified *K. pneumoniae* isolates (KP45 and KP57) as belonging to sequence type ST14, a lineage frequently associated with multidrug resistance and high pathogenicity (Table 4). These isolates exhibited resistance to a wide range of antibiotics, including beta-lactams (ampicillin, amoxicillin–clavulanic acid, and piperacillin–tazobactam), cephalosporins (cephalothin, cefoxitin, ceftazidime, and ceftriaxone), and carbapenems (meropenem and imipenem). Additionally, they were resistant to fluoroquinolones (ciprofloxacin), nitrofurantoin, and trimethoprim. Resistance was also observed against aminoglycosides, including amikacin, with all ST14 isolates exhibiting resistance to tigecycline. However, KP45 demonstrated intermediate resistance to gentamicin.

The *P. aeruginosa* isolate (PA85) was identified as sequence type ST233, a globally prevalent MDR lineage (Table 4). This isolate displayed resistance to all tested antibiotics, including aminoglycosides, carbapenems, fluoroquinolones, and cephalosporins. Susceptibility was only observed for colistin, consistent with its role as a last-resort treatment for MDR *P. aeruginosa*.

WGS analysis also revealed key resistance genes in the sequenced isolates. In *K. pneumoniae*, aminoglycoside resistance correlated with the presence of genes encoding aminoglycoside-modifying enzymes (AMEs), such as *AAC(6′)-Ib9*, *AAC(6′)-Ib-cr*, *aadA2*, and *ANT(3″)-Iia*. *AAC(6′)-Ib9* and *AAC(6′)-Ib-cr* encode aminoglycoside acetyltransferases located on plasmid transposon integrons within Enterobacteriaceae, whereas *aadA2* and *ANT(3″)-Iia* encode nucleotidyltransferases. Additionally, an *acrD* efflux pump and the *omp* gene were identified, further contributing to resistance mechanisms.

In *P. aeruginosa*, efflux pump genes such as *MexA*, *MexB*, *MexN*, *MexQ*, and *MexP*, along with the *mexAB-oprM* efflux pump regulator, were detected. Multiple outer membrane protein genes, including *oprM*, *oprN*, and *oprJ*, were also identified. These genetic determinants align with the observed phenotypic resistance patterns, highlighting the multidrug resistance mechanisms in these isolates.

## 3. Discussion

Antibiotic misuse contributes to the emergence of antimicrobial-resistant isolates in humans, animals, and the environment, exacerbating the global health crisis of antibiotic resistance, a significant public health threat in the 21st century [5].

Emerging evidence suggests that nanomaterials loaded with antibiotics can enhance antibiotic concentration at infection sites and promote liposome–bacterium interactions [19]. Encapsulation of antibiotics within lipid vesicles has been shown to reduce toxicity and improve efficacy against pathogens, while target selectivity may help overcome antibiotic resistance [19]. This study investigated whether a liposomal formulation combining TOB and MGO, a derivative of MH, could enhance TOB efficacy against bacteria. The antibiofilm activity of the liposomal formulation and its ability to prevent bacterial adhesion to the lung epithelial cell line A549 were evaluated. Additionally, the liposomal formulation was characterized by analyzing its morphology (assessed using dynamic light scattering and transmission electron microscopy [TEM]) and stability (assessed by calculating TOB retention under different biological conditions).

Liposomes can be classified based on their size into small (≤100 nm), intermediate (100–250 nm), large (≥250 nm), and giant (>1 μm) [20]. Liposome size is critical for stability, encapsulation efficiency, biodistribution, mucoadhesion, and cellular uptake [21]. It also influences the interaction between liposomes and bacterial biofilms, as well as the drug release pattern [22]. Bacterial cells typically have an average size of 1 μm, and smaller liposomes can fuse more closely with bacterial membranes, enhancing the release of encapsulated drugs [23]. Zhu et al. investigated the antibiofilm properties of gentamicin-loaded liposomes of various sizes (0.1–5.0 μm) against Gram-positive isolates, reporting maximum antibacterial activity with liposomes around 850 nm in size [24]. Nanoscale particles with aerodynamic diameters below 1 μm, such as the current liposomal formulation (580 nm), can effectively reach deep lung regions, including the alveoli, where *P. aeruginosa* infections often occur. Additionally, larger particles in the microscale range can distribute throughout the bronchial–alveolar region via sedimentation and Brownian motion, as shown in studies on lipid nanoparticle formulations [25]. The current Lip-MGO-TOB liposomes were within the acceptable size range for delivering encapsulated antibiotics to the lower airway, whereas nanoparticles larger than 5 μm are more likely to be trapped in the upper airway [26]. The PDI of the liposomal formulation indicated size heterogeneity. A PDI value closer to 0.0 represents a more uniform particle size population, whereas values exceeding 0.7 indicate greater size variability [23].

Liposome stability and drug retention are critical for developing effective drug nanodelivery systems [24]. The Lip-MGO-TOB formulation, composed of the saturated lipids 1,2-distearoyl-sn-glycero-3-phosphocholine (DSPC, neutral charge) and 1,2-bis(diphenylphosphino)ethane (DPPE, negatively charged), both with high transition temperatures (55 °C and 63 °C, respectively), along with cholesterol, likely contributed to its high drug retention under various conditions [6]. Liposome stability is often assessed by the magnitude of the zeta potential, with values between −30 mV and +30 mV considered indicative of stable formulations [27]. The zeta potential of the empty liposomes was −20 mV, which shifted to +26 mV after encapsulating MGO and TOB, indicating that the formulation was relatively stable and aligned with the observed stability results. This change in charge may be attributed to alterations in the fluidity of the lipid membrane, which can influence liposomal stability [28].

The Lip-MGO-TOB formulation displayed a classical liposomal morphology with a characteristic spherical shape within the accepted nanoscale range [29]. Its properties, including a high encapsulation efficiency of 83%, likely contributed to the enhanced antibacterial and antibiofilm activity of TOB against MDR isolates. A previous study that developed liposomal TOB (400 nm) demonstrated that the particles were electrostatically immobilized near biofilm clusters and were able to penetrate the biofilm matrix, enhancing their efficacy [30].

WGS data provided insights into the genomic structure of the bacterial isolates, enabling the identification of their resistance mechanisms. Sequencing data analysis revealed the presence of efflux pumps, outer membrane proteins, antibiotic-modifying enzymes, and biofilm formation genes. The resistance–nodulation–division superfamily of multidrug efflux pumps contributes to both intrinsic and acquired multidrug resistance by actively extruding antibiotics. These pumps also play roles in bacterial stress responses and pathogenicity [31]. Efflux pumps work synergistically with the outer membrane permeability barrier to enhance resistance by expelling harmful agents from bacterial cells [31]. Furthermore, 90% of naturally occurring biofilm-forming bacteria utilize efflux pumps, which significantly influence biofilm development and contribute to persistent infections [32]. Disrupting the MexAB–OprM efflux complex has been shown to increase hypersensitivity in *P. aeruginosa* to various antimicrobial agents [33]. Based on WGS data analysis, *P. aeruginosa* was found to possess multiple outer membrane protein genes such as *oprM*, *oprN*, and *oprJ*, as well as efflux pump genes, including *MexA*, *MexB*, *MexN*, *MexQ*, and *MexP*, which contribute to its resistance to multiple antimicrobial agents [34] and bacterial quorum-sensing signaling [35] (Supplementary Data Table S1). The low outer membrane permeability of *P. aeruginosa* was attributed to the slow porin *OprF* [31]. These resistance genes align with the antimicrobial susceptibility profiles listed in Table 2, confirming the resistance of *P. aeruginosa* to TOB. *K. pneumoniae* isolates were found to harbor the *acrD* efflux pump, likely explaining their high resistance to TOB. The *omp* gene, which plays a critical role in adherence, invasion, and biofilm formation, was also identified. Additionally, AME genes, such as *AAC(6′)-Ib9* and *AAC(6′)-Ib-cr*, were detected. These genes encode aminoglycoside acetyltransferases, which are carried on plasmid transposon integrons within Enterobacteriaceae. The nucleotidyltransferase genes *aadA2* and *ANT(3″)-IIa*, encoded on plasmids, were also present in all isolates (Supplementary Data Table S1). The prevalence of AME genes, including in Middle Eastern countries, limits the effectiveness of conventional aminoglycosides [36]. Further studies should focus on investigating the mechanisms by which liposomal formulations interfere with bacterial resistance pathways and their potential role in overcoming these challenges.

This study successfully developed a Lip-MGO-TOB system and evaluated the antibacterial activity of encapsulated TOB against MDR bacteria. Previous research has demonstrated the synergistic effect of MGO with amikacin against *Mycobacterium abscessus*, highlighting its potential to enhance the activity of aminoglycoside antibiotics [37]. In our study, free MGO inhibited bacterial growth at low concentrations (18–36 µg/mL). MGO exhibits bactericidal activity by interfering with protein synthesis through interactions with guanine residues in DNA, RNA, and their precursors within pathogenic bacterial cells [38]. Additionally, MGO can disrupt structural and regulatory genes, such as those associated with flagella and pili, further contributing to its antibacterial effects [39]. Future research should explore the impact of MGO on prevalent resistance mechanisms to better understand its potential as an adjunctive antibacterial agent. To the best of our knowledge, this is the first study to incorporate MGO into a liposomal formulation alongside TOB.

The clinical isolates evaluated in this study demonstrated high resistance to TOB. For *P. aeruginosa* isolate PA85, the MIC of free TOB was 64 µg/mL, which decreased to 32 µg/mL with the Lip-MGO-TOB formulation. *K. pneumoniae* isolates exhibited significant resistance to TOB, likely due to the presence of one or more TOB-modifying enzymes. Isolates KP45 and KP57 showed resistance at an MIC of 1024 µg/mL, consistent with the detection of AME genes in both isolates. However, the Lip-MGO-TOB formulation reduced the MIC for these resistant isolates to 512 µg/mL. The presence of *armA* (aminoglycoside-resistant methyltransferase) and *AAC(6′)-Ib-cr* genes in the tested *K. pneumoniae* isolates likely contributed to TOB resistance. The observed reduction in MIC values aligns with findings from previous studies that incorporated various antibiotics into liposomal formulations [40,41]. Additionally, Lip-MGO-TOB exhibited a significant antibiofilm effect against TOB-resistant isolates compared to free TOB. Liposomes are particularly effective in combating biofilms due to their ability to encapsulate and protect drugs from degradation while providing sustained release [42]. Their nanoscale size enables penetration into the biofilm matrix, and their lipid bilayer structure mimics bacterial membranes, facilitating fusion and efficient drug delivery. Furthermore, liposomal formulations can help overcome resistance by reducing the effective dose required, ensuring bacterial eradication before resistance can develop [43].

Adherence is the initial step in infection when bacteria attach to their target host cells [44]. In this study, the Lip-MGO-TOB formulation reduced the adhesion activity of *K. pneumoniae* isolate KP57 to A549 epithelial cells at a lower concentration (256 µg/mL) compared to TOB alone (512 µg/mL). Adherent bacteria can evade host immunity, develop resistance, and progress to chronic infections [45]. Therefore, preventing bacterial adhesion during the early stages of infection is critical for managing pulmonary infections.

The enhanced antimicrobial activity of the liposomal formulation may be attributed to its interaction with the bacterial outer membrane [6]. The structural similarity between bacterial membranes and liposomes likely facilitates unique interactions, promoting liposome–bacteria fusion, enabling efficient antibiotic delivery, and effectively targeting resistant isolates [5]. Additionally, the incorporation of MGO with TOB likely contributes to the observed antibacterial and antibiofilm activity. Both MGO and TOB interfere with bacterial protein synthesis [2,38], potentially exerting a synergistic effect against resistant isolates.

## 4. Materials and Methods

### 4.1. Preparation of Lip-MGO-TOB

Liposomes were prepared using the thin-film hydration method, as described by Mugabe et al., with modifications [46]. DSPC (purity > 99%), cholesterol (purity ≥ 99%), and DPPE (purity > 98%) (UFC Biotechnology, New York, NY, USA) were mixed in a molar ratio of 1.8:1:1 and dissolved in 1 mL of an ethanol:chloroform solution in a 50 mL round-bottom flask. MH was added as a surfactant at a weight ratio of 0.25:1 [13]. The lipid film was formed using a rotary evaporator at 65 °C under a controlled vacuum, followed by the removal of excess organic solvents with nitrogen gas. The lipid film was then rehydrated with distilled water (dH_2_O) containing 1 mg/mL of TOB and 0.4 mg/mL of MGO in a water bath at 65 °C with minimal light exposure. The formulation was subsequently sonicated using an ultrasonication probe for 3 min with 5-s on/off pulses at 60% intensity, repeated three times. Finally, the Lip-MGO-TOB formulation was lyophilized under the following conditions: vacuum 0.100 mbar; shelf temperature −40 °C; and collector temperature −80 °C for 72 h (LAB-CONCO FreeZone Freeze Dryers, Labconco, Kansas City, MO, USA). The resulting powdered formulation was stored at 4 °C for further use.

For the rehydration step, dH_2_O was added gradually (20% of the final volume) to the dried powdered formula, followed by vortexing and incubation for 15 min at 50 °C. This process was repeated thrice until the final volume of 2 mL was reached. Finally, the formulation was centrifuged at 10,000 rpm for 30 min to remove unencapsulated TOB, repeating the centrifugation step thrice.

### 4.2. Characterization of Lip-MGO-TOB

#### 4.2.1. Size and Zeta Potential Determination of the Liposomal Formulation

The mean diameter of Lip-MGO-TOB was measured using the laser light scattering technique (Malvern Zetasizer Nano, Malvern Panalytical, Malvern, UK, model ZEN5600). The liposomes were diluted in double dH_2_O and loaded into a transparent glass cuvette. The measurement process was repeated thrice to obtain the average liposome size [47]. The zeta potential of both the empty liposomes and the encapsulated liposomal formulation was also recorded for comparison. For this, 20 µL of the liposomes was diluted with 1000 µL of dH_2_O and tested using disposable folded capillary cells (Malvern DTS1070).

#### 4.2.2. Assessment of the EE and DLC of Lip-MGO-TOB

A well-diffusion assay was performed to determine the concentration of the encapsulated TOB, as described by Alhariri et al., with modifications [48]. A twofold serial dilution of 1.5× the initial TOB concentration was prepared. After rehydration and triple centrifugation of the Lip-MGO-TOB formulation, the supernatant was collected and replaced with dH_2_O after each centrifugation step. A 0.5 McFarland standard of *Escherichia coli* ATCC 29522 was prepared and streaked onto Mueller–Hinton agar (MHA) plates. Wells of 6 mm diameter were created in the agar and loaded with 50 µL of the collected supernatant and the antibiotic serial dilutions. The average of triplicate measurements was used for data analysis. The plates were incubated at 37 °C for 24 h. A standard curve (R^2^ = 0.9948) was generated using the TOB concentration versus the corresponding inhibition zone diameter. The EE% and DLC% of Lip-MGO-TOB were calculated as follows [49]:

$$\mathrm{EE}\%=\frac{(\mathrm{Intial}\;\mathrm{TOB}\;\mathrm{concentration}-\mathrm{Unloaded}\;\mathrm{TOB}\;\mathrm{concentration})\times 100}{\mathrm{Intial}\;\mathrm{TOB}\;\mathrm{concentration}}$$

$$\mathrm{DLC}\%=\frac{\mathrm{Encapsulated}\;\mathrm{TOB}\;\mathrm{concentration}(\mathrm{Lip}-\mathrm{MGO}-\mathrm{TOB})\times 100}{\mathrm{Intial}\;\mathrm{TOB}\;\mathrm{concentration}+\mathrm{Concentration}\;\mathrm{of}\;\mathrm{excipients}}$$

#### 4.2.3. Morphology Assay of Lip-MGO-TOB

TEM was performed to evaluate the morphology of the liposomes [50]. Briefly, 10 µL of diluted liposome solution was placed on a strip of parafilm. A formvar/carbon-coated 200-mesh copper grid was placed onto the liposome drop and left for 5 min to allow for adsorption. The grid was then transferred onto a drop of uranyl acetate stain for an additional 5 min. The excess stain was removed by gently dabbing the grid with an adsorbent pad. TEM images were captured using a JEM-1400 instrument (JEOL JEM-1400, Peabody, MA, USA) to visualize the liposomal morphology [50].

#### 4.2.4. Stability of Lip-MGO-TOB Under Different Biological Conditions

A stability assay was performed to evaluate the ability of Lip-MGO-TOB to retain TOB by calculating the percentage of TOB retained within the liposomal formulation [51]. The retention rate of antibiotics within the Lip-MGO-TOB formulation was assessed in PBS at 4 °C and 37 °C. Additionally, the Lip-MGO-TOB formulations were incubated in plasma and sputum at 37 °C to simulate biological conditions. The sputum sample was voluntarily provided by a 30-year-old healthy man, diluted 1:10 (*w*/*v*) in sterile PBS, and autoclaved at 120 °C for 15 min. Plasma samples were obtained anonymously from the hematology laboratory at King Abdulaziz Medical City (KAMC), Riyadh, after routine screening and prior to disposal. Briefly, 100 µL of the Lip-MGO-TOB formulation was combined with 100 µL of each test condition in triplicate with mild agitation (100–120 rpm). The samples were incubated at specific intervals (0, 1, 3, 6, 12, and 24 h) [26]. Following incubation, the supernatants were collected to determine the released antibiotic concentration using a well-diffusion assay and the following equation [51]:

$$Retention\;of\;the\;encapsulated\;drugs=\frac{initial\;concentration-released\;concentration}{initial\;concentration}\times 100$$

### 4.3. Biological Assays

#### 4.3.1. Bacterial Isolates and Susceptibility Tests

Clinical isolates of *P. aeruginosa* and *K. pneumoniae* were obtained anonymously from the Infectious Diseases Research Department at the King Abdullah International Medical Research Center and the Pathology and Laboratory Medicine Department (KAMC) following routine laboratory procedures. These isolates were selected based on their multidrug resistance profiles. All isolates were cultured in Mueller–Hinton broth and preserved in 25% glycerol stocks at −80 °C. Antibiotic susceptibility testing (AST) was performed using the VITEK 2 Compact automated system (bioMérieux, Craponne, France). Gram-negative identification cards were used for bacterial identification, whereas AST-N291 and AST-N292 cards were employed for antibiotic susceptibility testing of Gram-negative bacilli. All antibiotics and chemicals used in this study were procured from local vendors.

#### 4.3.2. Determination of TOB MIC Using the Microbroth Dilution Method

##### Inoculum Preparation

Sterile loops (1.0 µL) were used to streak bacterial isolates from glycerol stocks onto MHA (Scharlab Company, Barcelona, Spain) plates. The plates were incubated at 37 °C for 24 h. A stock solution of TOB (5000 µg/mL) was prepared in sterile water. All tested bacterial isolates were adjusted to 0.1 McFarland standard using DensiCHEK (bioMérieux, Minato City, Japan), as described by Eduardo et al. [52].

##### Determination of MICs of TOB, MGO, and Lip-MGO-TOB

The broth microdilution method was employed to determine the minimum inhibitory concentrations (MICs) of TOB and the Lip-MGO-TOB formulation. The method was validated according to the European Committee on Antimicrobial Susceptibility Testing breakpoints [53]. Briefly, 100 µL of Mueller–Hinton broth was added to each well of a 96-well plate, except for the first well. A 200 µL aliquot of the tested drug or formulation, prepared at twice the target concentration (2×), was added to the first well. Serial dilutions were performed by transferring 100 µL of the solution from one well to the next and discarding 100 µL from the last well. Subsequently, 100 µL of the adjusted bacterial inoculum was added to each test well, along with a positive control well containing 100 µL of Mueller–Hinton broth and the inoculum. Mueller–Hinton broth alone served as the negative control. *E. coli* ATCC 25922 was used as the quality control strain for AST [53,54].

##### Determination of Synergism Between TOB and MGO via Checkerboard Assay

The synergistic interaction between TOB and MGO was assessed using the checkerboard broth microdilution method and FICI calculation, as previously described [55]. Briefly, twofold serial dilutions of TOB (ranging from 1024 to 2 µg/mL) were prepared along the horizontal rows of a microtiter plate. These were cross-diluted vertically with twofold serial dilutions of MGO (ranging from 312 to 0.30 µg/mL). Subsequently, 100 µL of the prepared *P. aeruginosa* inoculum (adjusted to 0.5 McFarland standard) was added to each well. The plates were incubated at 37 °C for 24 h, after which the MICs were visually recorded.

#### 4.3.3. Antibiofilm Assays

The antibiofilm activity of the Lip-MGO-TOB formulation against resistant bacterial isolates was evaluated using a previously described method with modifications [56]. An overnight bacterial culture, adjusted to an optical density (OD_600_ nm) of 0.05, was used. The samples were seeded into a 96-well polystyrene plate with sub-MIC concentrations of the Lip-MGO-TOB formulation and free TOB. The plates were incubated at 37 °C for 24 h. After incubation, unattached (planktonic) bacteria were removed by washing the plates with sterile dH_2_O. The plates were dried in an oven at 50 °C for 45 min, followed by staining with 0.1% crystal violet for 15 min. Excess stain was removed, and the plates were washed thrice with PBS. Subsequently, 200 µL of 95% ethanol was added to each well to solubilize the stain for 15 min. The OD_600_ value was measured using a microplate reader [56]. The positive control consisted of untreated biofilms without any drug formulations, whereas the negative control contained only sterilized fresh media without bacterial or drug additives.

#### 4.3.4. Lip-MGO-TOB Activity Against Bacterial Adhesion to Human Cell Assays

The ability of the Lip-MGO-TOB formulation to prevent bacterial adhesion to A549 human carcinoma epithelial cells (ATCC CCL-185™, Manassas, VA, USA) was assessed, as adhesion is a critical step in biofilm formation [44]. A total of 1 × 10^6^ A549 cells were seeded into each well of six-well tissue-culture plates and incubated overnight at 37 °C in a 5% CO_2_ incubator. The cells were cultured in Dulbecco’s Modified Eagle Medium supplemented with 10% (*v*/*v*) heat-inactivated fetal bovine serum (Gibco^TM^, Grand Island, New York, NY, USA), 1% streptomycin, and 1% glutamine. Before this experiment, cells were subcultured in media without streptomycin. At 70% confluence, sub-MIC concentrations of the Lip-MGO-TOB formulation and free TOB were added to the wells. The plates were then exposed to pathogenic bacteria (pre-biofilm stage) at a multiplicity of infection of 10 for 3 h at 37 °C in a 5% CO_2_ atmosphere [57]. Nonadherent bacteria were removed by gently washing the wells five times with PBS.

To detach the A549 cells, ethylenediaminetetraacetic acid was added, and the cells were transferred to tubes containing 5 mL of PBS and incubated at 4 °C for 10 min to release the bacteria. Bacterial counts were determined using the drop plate method on Luria–Bertani agar after performing tenfold serial dilutions in sterile PBS. After incubation at 37 °C for 24 h, colony-forming units (CFU/mL) were calculated. The positive control consisted of A549 cells exposed to bacteria without any treatment, whereas the negative control contained A549 cells without bacteria or drugs [44].

### 4.4. WGS

WGS data for the clinical isolates *K. pneumoniae* and *P. aeruginosa* were obtained from the Infectious Diseases Research Department at KAIMRC. DNA was extracted using the MagnaPure Compact System (Roche, Basel, Switzerland). The DNA library was prepared using the Nextera XT Library Prep Kit (Illumina, San Diego, CA, USA), and short-read sequences were generated using the Illumina MiSeq System (Illumina, USA). FASTA file formats containing the complete nucleic acid sequences of the representative clinical isolates were retrieved for further analysis.

### 4.5. MLST and Detection of Resistance and Virulence Genes

MLST was employed as a molecular typing technique to determine the sequence type of the clinical isolates. This method is based on sequencing internal fragments of multiple housekeeping genes, typically between 7 and 11 loci. Each isolate was characterized by a set of seven numbers, each corresponding to a specific housekeeping allele [58].

Resistance genes, efflux pumps, outer membrane proteins, and biofilm formation genes were manually identified using CLC Workbench Version 8.1.3 (Qiagen, Aarhus, Denmark). Reference genes were obtained from the NCBI database, and detection was performed by aligning the reference genes with the whole genome of each isolate. Antimicrobial resistance genes and virulence factor genes were further analyzed using ABRicate (version 0.9.8; Seemann T, GitHub: https://github.com/tseemann/abricate, accessed on 17 January 2022) with the Megares database [59], ResFinder version 3.2, accessed on 15 November 2021) [60], and the virulence factor database [61]. 

### 4.6. Statistical Analysis

All statistical analyses were performed using Microsoft Excel and GraphPad Prism Version 5.0. The means and standard deviations of triplicate experiments were calculated for all assays. Data were analyzed using a one-way analysis of variance, followed by Tukey’s multiple comparison test.

## 5. Conclusions

This study highlighted the potential of a novel liposomal formulation containing MGO to enhance the efficacy of TOB against MDR bacteria. The Lip-MGO-TOB formulation achieved a twofold reduction in TOB’s effective dose (MIC) despite the presence of virulence factors and resistance mechanisms. The incorporation of MGO within the liposomes likely disrupted bacterial protein synthesis, potentially working synergistically with TOB to inhibit biofilm formation and reduce bacterial adhesion to A549 cells. The physicochemical properties of the Lip-MGO-TOB formulation make it a promising treatment option for combating TOB-resistant isolates. Further studies should prioritize evaluating the cytotoxicity of this formulation across various cell lines and animal models. Considering that inhaled TOB is widely used to treat pulmonary infections in cystic fibrosis patients, this formulation could be optimized for nebulizer applications, providing a targeted and effective approach against respiratory tract infections.

## Figures and Tables

**Table 1:** Characteristics of Lip-MGO-TOB liposomes.

Size (nm ± SD)	582.2 ± 44.80
PDI	0.52
Zeta potential of empty liposomes (mV ± SD)	−20.9 ± 1.39
Zeta potential of Lip-MGO-TOB liposomes (mV ± SD)	+26.1 ± 0.80
Entrapped concentration (mg/mL)	0.83
Encapsulation efficiency (%)	83.4
Drug loading capacity (%)	6.3

Size and zeta potential measurements represent the mean of triplicates ± SD. Abbreviations: Lip-MGO-TOB, liposomal methylglyoxal–tobramycin formulation; SD, standard deviation; PDI, polydispersity index.

**Table 2:** Antimicrobial susceptibility profile of the tested clinical isolates.

Antibiotic	Isolate ID (Source)					
	PA85 (Respiratory)		KP57 (Urine)		KP45 (Tissue)	
	MIC	ASTR	MIC	ASTR	MIC	ASTR
TIM	≥128	R	NA	NA	NA	NA
CAZ	≥64	R	≥64	R	≥64	R
FEP	≥64	R	≥64	R	≥64	R
IMP	≥16	R	≥16	R	≥16	R
MEM	8	R	≥16	R	≥16	R
AMK	≥64	R	≥64	R	≥64	R
GEN	≥16	R	8	I	≥16	R
TOB	≥16	R	NA	NA	NA	NA
CIP	≥4	R	≥4	R	≥4	R
LVX	≥8	R	NA	NA	NA	NA
TGC	≥8	R	≥8	R	≥8	R
CST	≥0.5	S	NA	NA	NA	NA
AMP	NA	NA	≥32	R	≥32	R
AMC	NA	NA	≥32	R	≥32	R
PIP/TAZ	≥128	R	≥128	R	≥128	R
CF	NA	NA	≥64	R	≥64	R
FOX	NA	NA	≥64	R	≥64	R
CRO	NA	NA	≥64	R	≥64	R
NIT	NA	NA	256	R	256	R
TMP	NA	NA	NA	R	NA	R
TMP-SMX	NA	NA	≥320	R	≥320	R
Resistance score	26		30		32	

Abbreviations: TIM, Ticarcillin–clavulanic acid; CAZ, Ceftazidime; FEP, Cefepime; IMP, Imipenem; MEM, Meropenem; AMK, Amikacin; GEN, Gentamicin; TOB, Tobramycin; CIP, Ciprofloxacin; LVX, Levofloxacin; TGC, Tigecycline; AMP, Ampicillin; AMC, Amoxicillin–clavulanic acid; PIP/TAZ, Piperacillin–tazobactam; CF, Cephalothin; FOX, Cefoxitin; CRO, Ceftriaxone; NIT, Nitrofurantoin; TMP, Trimethoprim; TMP-SMX, Trimethoprim–sulfamethoxazole; CST, Colistin; ASTR, Antimicrobial susceptibility testing result; MIC, Minimum inhibitory concentration; R, Resistant; S, Susceptible; I, Intermediate; NA, Not available.

**Table 3:** MICs and MBCs of TOB and Lip-MGO-TOB against the tested clinical isolates.

Bacteria	TOB (µg/mL)		Lip-MGO-TOB (µg/mL)	
	MIC	MBC	MIC	MBC
KP45	1024	2048	512	1024
KP57	1024	2048	512	1024
PA85	64	128	32	64
*Escherichia coli* ATCC 25922	2	2	-	-

Abbreviations: MIC, Minimum inhibitory concentration; MBC, Minimum bactericidal concentration; TOB, Tobramycin; Lip-MGO-TOB, Liposomal methylglyoxal–tobramycin formulation; KP, *Klebsiella pneumoniae*; PA, *Pseudomonas aeruginosa*.

**Table 4:** Locus numbers for each housekeeping gene and sequence types of the clinical isolates.

Isolate ID (Accession No.)	*gapA*	*infB*	*mdh*	*pgi*	*phoE*	*rpoB*	*tonB*	*ST*
KP45 (SAMN45105960)	1	6	1	1	1	1	1	14
KP57 (SAMN45105961)	1	6	1	1	1	1	1	14
**Isolate ID**	***acsA***	***aroE***	***guaA***	***mutL***	***nuoD***	***ppsA***	***trpE***	***ST***
PA85 (SAMN20514487)	16	5	30	11	4	31	41	233

## Data Availability

The genome data generated in this study have been deposited in the NCBI database under BioProject PRJNA1192405. The specific BioSample details are as follows: *Pseudomonas aeruginosa* isolate PA85 (BioSample SRR15523612), *Klebsiella pneumoniae* isolate KP45 (BioSample SAMN45105960), and *Klebsiella pneumoniae* isolate KP57 (BioSample SAMN45105961). These datasets are publicly accessible via the NCBI BioProject database at https://www.ncbi.nlm.nih.gov/bioproject/PRJNA1192405, accessed on 2 December 2024.

## Supplementary Materials

The following supporting information can be downloaded at: https://www.mdpi.com/article/10.3390/antibiotics14010003/s1, Table S1. Heat-map of aminoglycosides modifying enzymes (AMEs) in *K. pneumonia* and *P. aeruginosa* clinical isolates.
