# Supplementary material for: Effectiveness of a Novel Liposomal Methylglyoxal–Tobramycin Formulation in Reducing Biofilm Formation and Bacterial Adhesion
Source: Antibiotics (Basel). 2024 Dec 24;14(1):3. doi: 10.3390/antibiotics14010003 (PMC11763214; doi:10.3390/antibiotics14010003)
Supplement: Supplementary file 1 [file antibiotics-14-00003-s001.zip › antibiotics-3310835-supplementary.pdf]

Supplementary Table S1. Heat-map of aminoglycosides modifying enzymes (AMEs) in *K. pneumonia* and *P. aeruginosa* clinical isolates.

**Isolates ID**	**Aminoglycosides modifying enzymes (AMEs) genes**								
	**AAC(6')-Ib9**	**AAC(6')-Ib-cr**	**aadA2**	**acrD**	**ANT(3'')-IIa**	**APH(3'')-Ib**	**APH(3')-VI**	**APH(6)-Id**	**armA**
**KP_WGS/kb-45**		■	■	■	■	■	■	■	■
**KP_WGS/kb-57**		■	■	■	■	■	■	■	■

**Isolates ID**	**Aminoglycoside resistance genes**						
	**AAC(3)-Id**	**AAC(6')-Ib9**	**AAC(6')-II**	**aadA2**	**aadA6**	**ANT(3'')-IIa**	**APH(3')-IIb**
**P.aeruginosa/RPA85**	■		■	■			■

The black highlight (■) indicates the presence of the gene.
